# Green-Synthesized Zinc Oxide Nanoparticles for Acinetobacter baumannii Control: A Review of Plant-Based Approaches

**DOI:** 10.7759/cureus.83506

**Published:** 2025-05-05

**Authors:** Omar I Khalif, Esam J Alkalifawi

**Affiliations:** 1 Department of Biology, College of Education for Pure Science (Ibn Al-Haitham), University of Baghdad, Baghdad, IRQ

**Keywords:** acinetobacter baumannii, antimicrobial activity, green synthesis, plant-based nanotechnology, zinc oxide nanoparticles

## Abstract

*Acinetobacter baumannii* (*A. baumannii) *is an opportunistic pathogen responsible for severe nosocomial infections, often exhibiting multidrug resistance (MDR), which limits treatment options. As a promising alternative to conventional antibiotics, zinc oxide nanoparticles (ZnO NPs) have attracted significant attention due to their potent antimicrobial properties. In particular, plant-based biosynthesis of ZnO NPs offers an eco-friendly, cost-effective, and sustainable approach compared to chemical and physical synthesis methods. Phytochemicals reduce and stabilize agents in the synthesis of NPs, enhancing biocompatibility and minimizing toxic effects. This review explores the potential of biosynthesized ZnO NPs for controlling *A. baumannii* infections and highlighting their antimicrobial mechanisms. Furthermore, we discuss the advantages of plant-mediated synthesis and the challenges in clinical translation. Understanding the role of biosynthesized ZnO NPs in combating MDR pathogens could pave the way for novel therapeutic strategies against *A. baumannii* infections.

## Introduction and background

The World Health Organization (WHO) has identified antimicrobial resistance as one of the top three global public health threats [1]. Among the most concerning contributors to this crisis are the ESKAPE pathogens, namely, *Enterococcus faecium*, *Staphylococcus aureus*, *Klebsiella pneumoniae*, *Acinetobacter baumannii *(*A. baumannii*), *Pseudomonas aeruginosa*, and *Enterobacter *spp., renowned for their ability to evade antibiotic treatments and cause severe, persistent infections [1,2]. Over the past few decades, *A. baumannii* has emerged globally as a highly significant opportunistic pathogen, frequently associated with healthcare-associated infections, particularly in critically ill and immunocompromised patients [2]. Its clinical importance stems from its extraordinary adaptability, allowing it to thrive in hospital environments, persist on medical equipment, and form biofilms, facilitating rapid nosocomial transmission. Infections caused by *A. baumannii*, such as ventilator-associated pneumonia (VAP), wound infections, and bloodstream infections, are associated with significant morbidity and mortality, particularly in intensive care units (ICUs), where mortality rates can exceed those observed in general hospital wards [3-5]. Of particular concern is *A. baumannii's* remarkable ability to acquire and upregulate diverse resistance determinants, enabling it to develop resistance to multiple antibiotic classes, including carbapenems, fluoroquinolones, and aminoglycosides. This has led to the emergence of multidrug-resistant (MDR), extensively drug-resistant (XDR), and even pan-drug-resistant (PDR) strains, posing a substantial challenge to global healthcare systems [6,7]. Consequently, *A. baumannii* is now considered one of the most formidable threats among nosocomial pathogens [2].

The alarming rise in antibiotic resistance highlights the urgent need for alternative therapeutic strategies to combat infections caused by *A. baumannii*. Among these strategies, nanotechnology has emerged as a promising avenue for antimicrobial innovation. Nanotechnology enables manipulating materials at the nanoscale (1-100 nm), enhancing their reactivity and functionality through an increased surface-area-to-volume ratio and quantum effects [8,9]. These properties make nanoparticles (NPs) highly effective in drug delivery, diagnostics, and antimicrobial treatments [10,11]. Metal-based NPs such as silver, titanium dioxide, and zinc oxide (ZnO) are particularly valued for their biocompatibility, chemical stability, and antimicrobial properties, making them essential in medicine, cosmetics, and electronics [12]. Their ability to generate reactive oxygen species (ROS) and interact with bacterial cell membranes contributes to their effectiveness in disrupting bacterial structures, inhibiting metabolic pathways, and inducing cell death [13-15]. ZnO NPs have garnered considerable attention among these NPs due to their biosafety profile, cost-effectiveness, and potent antimicrobial efficacy, making them suitable candidates for clinical and biomedical applications [15]. Recent advancements in the biosynthesis of ZnO NPs have further enhanced their potential for therapeutic applications. Unlike traditional chemical and physical synthesis methods, biosynthetic approaches utilize biological agents such as plant extracts, fungi, and bacteria, making them environmentally friendly and cost-effective. These green synthesis methods reduce toxic chemicals and produce NPs with improved stability and biocompatibility, essential features for biomedical applications [16].

This manuscript presents a narrative review aimed at synthesizing and discussing the current knowledge on the biological control of *A. baumannii* using biosynthesized ZnO NPs. It explores the mechanisms underlying their antibacterial activity, advances in their application, and the challenges and limitations of their use in clinical settings. By addressing both the advances and challenges, this review aims to provide a comprehensive understanding of the role of ZnO NPs in combating one of the most significant global healthcare threats.

## Review

*A. baumannii*: pathogenicity and antimicrobial resistance mechanisms

*A. baumannii* is recognized as a Gram-negative, non-motile, obligate aerobe, catalase-positive, oxidase-negative, and non-fermenting coccobacillus [17,18]. It was first isolated by Dutch microbiologist Martinus Beijerinck in 1911 from soil using minimal media enriched with calcium acetate. Initially, Beijerinck classified it as *Micrococcus calcoaceticus*. In 1954, Brisou and Prévot proposed the genus *Acinetobacter*, derived from the Greek word "akinetos," meaning "non-motile," to distinguish non-motile species from motile ones within the closely related genus *Achromobacter *[5,15]. The name *Acinetobacter *gained widespread acceptance in 1968 following the work of Baumann et al., who isolated multiple species from environmental sources such as soil and water [6,19,20]. In the early 2000s, *A. baumannii *emerged as a significant human pathogen, particularly among U.S. military personnel during the Iraq War. Its high prevalence in military hospitals earned it the colloquial name "Iraqibacter". This period marked a turning point in recognizing* A. baumannii *as a formidable nosocomial pathogen capable of causing severe infections such as pneumonia, bloodstream infections, and wound infections, particularly in critically ill patients [20]. *A. baumannii* has emerged as a significant nosocomial pathogen and is a critical global health concern. Its remarkable ability to persist in healthcare environments, its increasing resistance to multiple antimicrobial agents, and its ability to cause severe infections make it a formidable challenge in clinical settings [19,21]. This opportunistic pathogen is responsible for a broad spectrum of infections, including hospital-acquired infections (HAIs), VAP, bloodstream infections, urinary tract infections, meningitis, skin and soft tissue infections, burn and wound infections, endocarditis, and otitis media, particularly in immunocompromised patients in ICUs [20,22-25]. *A. baumannii* thrives in extreme conditions, tolerating temperature, pH, and nutrient availability fluctuations, which further reinforces its role as a significant pathogen in hospitals. Therefore, its resilience and adaptability make it particularly problematic in hospital settings, especially among immunocompromised individuals [6]. The pathogenicity of *A. baumannii* is multifaceted, involving various virulence factors that facilitate adhesion, invasion, immune evasion, and persistence. Additionally, its remarkable antimicrobial resistance mechanisms further complicate treatment, leading to high morbidity and mortality rates in healthcare settings. Understanding the molecular basis of its virulence and resistance is essential for developing new therapeutic strategies [26].

Virulence Factors of A. baumannii

Virulence factors in *A. baumannii *contribute to its ability to colonize, invade, and survive under stressful conditions, including antibiotic pressure. These factors include motility, outer membrane proteins (OMPs), biofilm formation, capsular polysaccharides (CPS), lipopolysaccharides (LPS), and protein secretion systems. Understanding these virulence mechanisms is crucial for developing effective therapeutic strategies against *A. baumannii* infections.

Motility and pili: Although *A. baumannii* lacks flagella, it exhibits surface-associated motility through type IV pili, essential for surface colonization, biofilm formation, and horizontal gene transfer [27,28]. The Csu pili system plays a major role in host tissue colonization and device attachment by promoting biofilm formation, even while indirectly reducing motility [29]. Structural studies highlight the role of the CsuE subunit’s hydrophobic projections in adherence to abiotic surfaces like plastics used in medical devices [30].

Outer membrane proteins: *A. baumannii*’s outer membrane provides a defensive barrier and contains various OMPs involved in virulence and drug resistance. Notable OMPs include OmpA, AbuO, CarO, OprD-like proteins, OprF, CadF, Omp22, Omp33-36, Oma87/BamA, NmRmpM, TolB, and DcaP [31-34]. These proteins form β-barrel channels for selective transport, regulate permeability, and restrict antibiotic entry [35]. OmpA is particularly well-studied, mediating adhesion via fibronectin binding and inducing apoptosis by mitochondrial targeting, releasing cytochrome c and AIF, and eventually leading to host DNA fragmentation [36,37]. OmpA is also secreted via outer membrane vesicles (OMVs), increasing its cytotoxic potential [38,39]. Additionally, it supports antibiotic resistance by interacting with efflux systems that extrude drugs like aztreonam and chloramphenicol [36]. Other OMPs also contribute significantly to *A. baumannii*'s virulence and antibiotic resistance. Proteins such as OccAB1-5, OmpW, and Omp33-36 are involved in nutrient uptake and host interaction. Omp33-36 is a water channel essential for host cell invasion, cytotoxicity, and apoptosis induction through caspase activation. It has been linked to the activation of mitochondrial ROS production, contributing to oxidative stress within host cells. This oxidative stress damages host cell components, including lipids, proteins, and DNA, which promotes tissue damage and immune evasion. Omp33-36 is associated with carbapenem resistance, as its absence has been correlated with increased resistance to carbapenems such as imipenem and meropenem [40]. Beyond its role in antibiotic resistance, Omp33-36 interferes with autophagy by increasing the expression of LC3B-II, an autophagosomal membrane protein, and p62/SQSTM1, a selective autophagy receptor. This disruption of the autophagic process allows *A. baumannii* to persist within autophagosomes, creating a protected niche for intracellular survival. The ability of Omp33-36 to modulate host cell death pathways, including autophagy and apoptosis, underscores its critical role in the bacterium’s virulence and persistence in host tissues [40]. CarO, a 25-29 kDa protein with subtypes CarOa and CarOb, is a crucial OMP in *A. baumannii* that facilitates the influx of β-lactam antibiotics, including carbapenems like imipenem, by allowing their passage through the outer membrane to reach periplasmic targets. This function is essential for the bactericidal activity of these antibiotics. In addition to its role in antibiotic transport, CarO has been implicated in immune modulation by downregulating the production of pro-inflammatory cytokines. This immune evasion strategy allows *A. baumannii* to persist within host tissues, contributing to chronic infections and immune system evasion. The dual role of CarO in antimicrobial resistance and immune modulation highlights its importance in the pathogenesis of MDR* A. baumannii* strains, particularly in hospital-acquired infections where carbapenem resistance presents a significant therapeutic challenge [34,41,42].

Biofilm formation: Biofilm formation in* A. baumannii *is a complex and dynamic process in which bacterial cells transition from a free-floating (planktonic) state to a sessile, surface-attached lifestyle. Various environmental factors influence this transition, including surface porosity, fluid dynamics, and nutrient availability. Biofilm development progresses through distinct stages: initial attachment, microcolony formation, maturation, and dispersal, with quorum sensing (QS) playing a pivotal role in regulating these processes [43]. Planktonic bacterial cells adhere to biotic or abiotic surfaces in the initial phase. This attachment is initially reversible, but as bacteria interact with the surface, they establish a more stable adhesion. Hydrophobic, nonpolar surfaces such as Teflon and certain plastics promote bacterial attachment more efficiently than hydrophilic surfaces like stainless steel, highlighting the role of hydrophobic interactions in overcoming repulsive forces [44]. Once a stable attachment is achieved, bacteria begin secreting extracellular polymeric substances (EPS), which reinforce adhesion and facilitate the formation of microcolonies. The proximity of bacterial cells within these microcolonies enables efficient nutrient exchange and the removal of metabolic by-products [43]. As the biofilm matures, it develops into an organized, three-dimensional structure shaped by nutrient availability. During this stage, bacterial cells communicate via QS, a signaling mechanism that regulates population density by releasing autoinducers. QS facilitates genetic adaptation, enhances resource utilization, and contributes to antibiotic tolerance within the biofilm. The EPS matrix also forms interstitial channels as a circulatory network, ensuring nutrient distribution and waste removal [45,46]. The final stage of biofilm development involves bacterial dispersal, allowing *A. baumannii *to colonize new environments. This transition from a sessile to a motile state is often triggered by environmental cues such as oxygen depletion, nutrient scarcity, or the enzymatic degradation of EPS components. Hydrolytic enzymes, including saccharolytic enzymes, degrade the matrix, enabling bacterial detachment and dissemination [43]. The regulation and stabilization of biofilms involve a complex interplay of genetic and environmental factors. Key determinants include the Csu pilus system [29], type IV pili [27], OMPs [34], QS [4], and the biofilm-associated protein (BAP) [47]. OMPs enhance bacterial adhesion to surfaces and contribute to biofilm resilience under adverse conditions [34]. QS, a crucial bacterial communication process, regulates gene expression in motility, biofilm formation, sporulation, and pathogenesis through small signaling molecules known as autoinducers, such as acyl-homoserine lactones (AHLs) [48]. *A. baumannii* produces 3-hydroxy-C12-homoserine lactone as its primary AHL and possesses a two-component QS system, AbaI-AbaR, which governs biofilm development and virulence [49]. The interaction of AHL with the AbaR receptor enhances csu gene expression, leading to increased Csu pilus production and biofilm formation [50]. Targeting QS pathways has emerged as a promising strategy to control *A. baumannii *infections. Several FDA-approved drugs, including erythromycin, chloroquine, streptomycin, and propranolol, have demonstrated the ability to inhibit QS pathways, providing potential avenues for disrupting biofilm formation and enhancing antimicrobial efficacy [51]. BAP is a crucial factor in* A. baumannii* biofilm formation, contributing to its adhesion, aggregation, and structural stability. As a high-molecular-weight surface protein, BAP is required to develop three-dimensional biofilm structures, including tower and water channel formation, facilitating nutrient exchange and waste removal. This protein is pivotal in enabling *A. baumannii* to persist on medically relevant surfaces such as polystyrene, polypropylene, and titanium, significantly increasing the risk of HAIs [52,53]. The ability of BAP to facilitate biofilm formation on medical devices highlights its importance in *A. baumannii *pathogenesis. Catheters, ventilators, and other medical implants serve as prime surfaces for biofilm development, leading to chronic infections that are highly resistant to conventional antibiotics [54]. Environmental factors tightly control the expression of the bap gene, with iron availability as a critical determinant. Studies have shown that bap expression is significantly upregulated under iron-limiting conditions, leading to enhanced biofilm formation and increased bacterial survival. This adaptation provides *A. baumannii* with a survival advantage in iron-restricted environments, such as within the human host or on medical devices, where iron sequestration by host proteins limits bacterial access to this essential nutrient [54]. BAP facilitates biofilm formation through multiple mechanisms. It is secreted via a Type I secretion system and promotes bacterial aggregation by interacting with host cell surface carbohydrates. During the initial stages of biofilm formation, BAP enhances adhesion to biotic and abiotic surfaces, ensuring stable bacterial attachment. BAP strengthens the biofilm matrix as it matures, reinforcing its structural integrity and increasing bacterial resilience against environmental stressors [55].

Capsule: The capsule is a significant virulence factor in *A. baumannii*, critical in its ability to evade the host immune system and enhance its pathogenicity. The bacterial capsule comprises repeating sugar units tightly linked to form a high-molecular-weight protective layer around the bacterial surface. This capsule includes various components such as CPS, lipooligosaccharides (LPS), and poly-β-(1-6)-N-acetylglucosamine exopolysaccharide (PNAG), which are high-molecular-weight hydrophilic polymers that envelope the bacterial cell and contribute to its resilience [56]. The CPS capsule serves multiple functions that aid in *A. baumannii'*s persistence and virulence. One of its key roles is reducing the interactions between the pathogen's surface structures and the host's immune defenses. By acting as a protective barrier, the capsule helps the bacterium evade immune recognition and resist complement-mediated bactericidal activity and serum components that would otherwise limit bacterial survival. This immune evasion is crucial for the persistence of *A*. *baumannii *in clinical settings, particularly in patients with compromised immune systems [56,57].

LPS and LOS: LPS and LOS are key virulence factors in *A. baumannii*, aiding immune evasion, structural stability, and antibiotic resistance. LPS consists of lipid A, a core oligosaccharide, and an O-antigen, whereas LOS lacks the O-antigen but retains lipid A and core oligosaccharide. LPS interacts with toll-like receptor 4 (TLR4), triggering inflammation and cytokine production. While intended to eliminate the pathogen, this response weakens neutrophil function, facilitating immune evasion. Modifications in LPS (such as lipid A acylation) enhance resistance to antimicrobial agents like polymyxins and β-lactams [2,58]. Similarly, LOS induces immune activation, impairing neutrophil activity and promoting bacterial persistence. The interaction of LPS, LOS, and CPS strengthens *A. baumannii’s* structure, aiding resistance to environmental stress and antimicrobials. Their role in biofilm formation further complicates treatment, contributing to persistent infections [56,59].

Evolution of antimicrobial resistance in *A. baumannii*: In the 1970s, *A. baumannii *strains were susceptible primarily to a broad range of antibiotics, including gentamicin, ampicillin, chloramphenicol, and nalidixic acid. However, the widespread and often indiscriminate use of broad-spectrum antibiotics in healthcare settings has driven the evolution of *A. baumannii* into a significant nosocomial pathogen, demonstrating resistance across multiple antibiotic classes [60,61]. Reports indicate that the increasing use of β-lactam antibiotics has played a substantial role in the emergence of drug-resistant *A. baumannii *strains. Initially, carbapenems were considered a primary treatment for MDR *A. baumannii *infections, but their frequent use has led to a significant rise in carbapenem resistance [62,63]. Currently, polymyxins are used for managing MDR *A. baumannii *infections despite concerns regarding their systemic toxicities, including nephrotoxicity and neurotoxicity. Strains resistant to aminoglycosides, cephalosporins, carbapenems, and fluoroquinolones are classified as XDR. Additionally, isolates that exhibit resistance to polymyxins and tigecycline are categorized as PDR [64]. The mechanisms contributing to *A. baumannii *resistance are complex and multifactorial, involving enzymatic degradation of antibiotics (such as β-lactamases and carbapenemases), overexpression of efflux pumps, modifications of target sites, permeability defects, aminoglycoside-modifying enzymes, and horizontal gene transfer of resistance determinants [6,65]. Efflux pumps, particularly those in the resistance nodulation-division (RND) family, play a significant role in* A. baumannii* resistance. These membrane proteins actively expel toxic compounds, including antibiotics, from the bacterial cell, reducing their efficacy. The rapid emergence of MDR, XDR, and PDR strains has significantly limited treatment options, posing a significant challenge in clinical settings. While colistin and tigecycline remain effective against MDR *A. baumannii,* the emergence of colistin-resistant strains worldwide presents a critical treatment challenge. Resistance to colistin is primarily associated with modifications to the bacterial cell envelope, such as the addition of phosphoethanolamine to lipid A, which reduces colistin's binding affinity. Additionally, LPS composition alterations contribute to colistin resistance [65-67]. Considering the increasing resistance to traditional antibiotics, alternative therapeutic strategies have gained attention. Recent strategies in antimicrobial peptides, iron chelation therapy, phage therapy, photodynamic therapy, nitric oxide-based treatments, and NP-based therapies have shown potential against *A. baumannii *infections. The continuous development of innovative approaches to combat MDR *A. baumannii* remains a critical priority in global healthcare research [66,68].

ZnO NPs: properties, synthesis, and applications

Zinc is an essential trace element and is the second most prevalent micronutrient in the human body. It plays a pivotal role in numerous biological processes, including cell proliferation, cell signaling, differentiation, and maintaining homeostasis. Zinc is also integral to immune function, oxidative stress responses, antioxidant activities, aging, and apoptosis as a cofactor in over 300 enzymes. Zinc is crucial for the structural organization of nucleic acids and in DNA replication and repair mechanisms [69].

ZnO is an inorganic zinc compound with a wide range of beneficial properties, making it an essential material in various industries such as medicine, cosmetics, and environmental engineering. ZnO is known for its inherent antibacterial, antifungal, and UV-absorbing properties. These characteristics make it a crucial ingredient in sunscreens and cosmetic formulations designed for UV protection. As an II-VI semiconductor, ZnO exhibits a wide direct band gap (3.3 eV) and high excitonic binding energy (60 meV), contributing to its excellent optical, electronic, and photocatalytic properties. ZnO's strong ionic bonding also ensures thermal and chemical stability, making it durable in different conditions [70]. At the nanoscale, ZnO transforms into ZnO NPs, which possess unique physical and chemical properties distinct from bulk ZnO. The high surface-area-to-volume ratio of ZnO NPs enhances their reactivity, increasing their effectiveness in antimicrobial applications. ZnO NPs are particularly notable for their ability to generate ROS, which are significant in their antibacterial and antifungal activities. These NPs are increasingly explored as potential agents to combat MDR pathogens, offering a promising alternative to traditional antibiotics [71,72].

ZnO NPs BioSynthesis: Plant-Based Sources

Recently, eco-friendly and biologically mediated strategies have gained significant attention for NP synthesis due to their cost-effectiveness and minimal environmental impact. The biological, or green, production of NPs employs biological agents, including microorganisms (bacteria, fungi, yeast, and algae) and plant extracts, and natural reducing and stabilizing agents, thereby eliminating the need for hazardous solvents and excessive energy input [73]. Critical factors such as pH, temperature, buffer selection, enzymatic activity, and biochemical pathways influence the efficiency and characteristics of the synthesized NPs. Furthermore, this method contributes to environmental sustainability by converting toxic metal waste into safer compounds, making it an effective alternative for large-scale production [74-76].

Plant-based NP synthesis is widely regarded as one of the simplest and most effective biological approaches. Numerous studies have successfully used plant extracts to synthesize ZnO nanomaterials that exhibit photocatalytic and antibacterial properties. Plant extracts contain various bioactive metabolites, including proteins, vitamins, coenzyme-based intermediates, phenolic compounds, flavonoids, and carbohydrates [77]. These substances play a crucial role in neutralizing free radicals, ROS, and chelating metal ions. Consequently, plant extracts act as both reducing agents and stabilizers during the synthesis of ZnO NPs [78]. The presence of reductive antioxidants, such as amino acids, polysaccharides, polyphenols, alkaloids, tannins, and saponins, alongside other bioactive compounds like terpenoids and flavonoids, promotes the reduction of Zn^2+^ ions in solution, resulting in the formation of stable, well-dispersed ZnO NPs [79]. Plant extracts can be employed in their aqueous form or concentrated, and they are mixed with zinc precursors under optimized pH and temperature conditions to achieve controlled NP formation. The basic mechanism behind this process involves phytochemicals acting as reducing and stabilizing agents, contributing to the controlled synthesis of ZnO NPs [80-82]. Distilled water and ethanol are the most commonly used solvents for preparing plant extracts, offering fewer health risks than microbial NP synthesis methods. Plant extracts derived from various plant parts, such as leaves, flowers, roots, barks, and peels, have all been utilized in ZnO NPs biosynthesis [83,84]. For instance, myrtle (*Myrtus communis*), an evergreen perennial plant native to North Africa and southern Europe, exemplifies the potential of plant-based synthesis [85]. It is characterized by its rich chemical composition, which includes compounds like tannins, sugars, volatile oils, and essential oils such as camphene, nerol, cineole, and Myrtol. These compounds are reducing agents in synthesizing ZnO NPs [86-88]. Additionally, myrtle fruits are rich in anthocyanins, phenols, and alkaloids, while their seeds contain glycerides such as palmitic and myristic acids. The leaves of myrtle also contain two essential antioxidant compounds, myrtucommulone A and myrtucommulone B, which enhance the antioxidant properties of the NPs produced. Due to its rich chemical composition and potent antioxidant activity, myrtle holds significant promise as a sustainable, low-cost source for the biosynthesis of ZnO NPs, making it an ideal candidate for green nanotechnology applications [89]. Similarly, algae, including species such as red algae (*Rhodophyceae*), green algae (*Chlorophyceae*), brown algae (*Phaeophyceae*), and blue-green algae (*Cyanophyceae*), have been recognized for their ability to produce metal and metal oxide NPs [90]. These organisms contain secondary metabolites like terpenoids, laminans, and fucoidans, which exhibit notable anti-cancer properties and can inhibit tumor growth. Importantly, algae do not require external reducing or capping agents, which makes them an energy-efficient, cost-effective, and environmentally friendly option for the production of NPs. The algae-based synthesis also holds promise for biomedical and pharmaceutical applications due to its natural bioactive compounds, which can contribute to the functional properties of the NPs [90-93]. Using plant-based methods for the synthesis of NPs offers several advantages, including imparting multifunctional properties, such as antimicrobial effects, without the toxicity commonly associated with chemically synthesized NPs. This makes plant-based NPs ideal for various applications, including biomedicine, biotechnology, and even textiles, where they can serve antimicrobial purposes [94].

**Figure 1:**
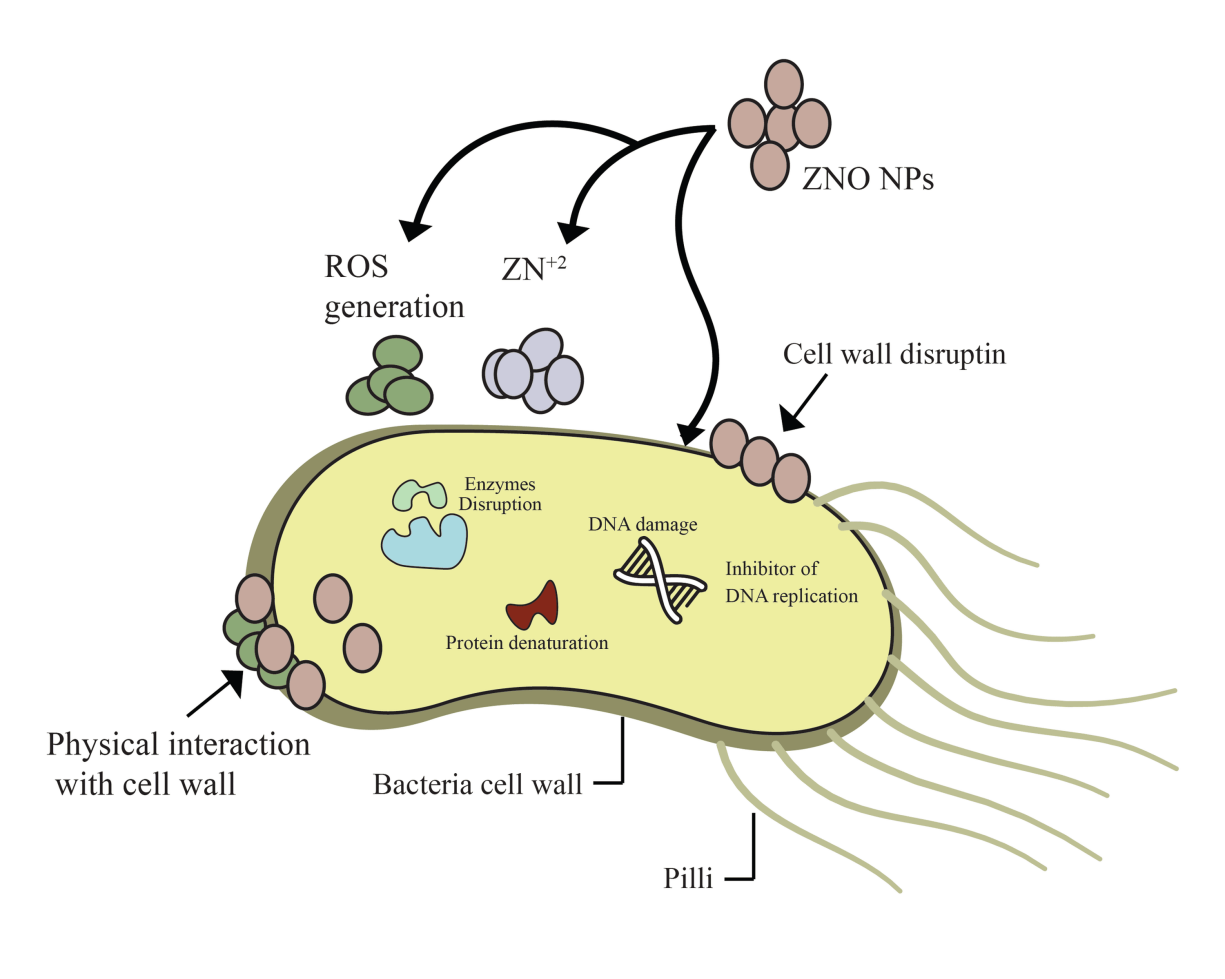
Antibacterial mechanism of zinc oxide nanoparticles (ZnO NPs) ZnO NPs exhibit antibacterial activity through multiple mechanisms: (1) Reactive oxygen species (ROS) generation: ZnO NPs produce hydroxyl radicals (•OH), superoxide anions (O₂•−), and hydrogen peroxide (H₂O₂), leading to oxidative stress, membrane damage, and disruption of essential biomolecules. (2) Zn²⁺ Ion Release: Dissolution of ZnO NPs results in the release of Zn²⁺ ions, which interfere with bacterial enzymatic functions and metabolic pathways, leading to cell death. (3) Internalization into bacterial cells: ZnO NPs penetrate bacterial cells, disrupting intracellular homeostasis and leading to oxidative stress and DNA damage. (4) Electrostatic interactions: positively charged ZnO NPs interact with the negatively charged bacterial membrane, causing membrane destabilization, increased permeability, leakage of intracellular contents, and bacterial lysis. These combined actions contribute to the strong antibacterial properties of ZnO NPs, making them potential candidates for combating multidrug-resistant (MDR) bacteria. Image credit: Omar I. Khalif; this figure was designed by the author and is unpublished.

Antimicrobial mechanisms of biosynthesized ZnO NPs: Studies demonstrated that green-synthesized ZnO NPs exhibit more potent inhibitory effects than their chemically synthesized counterparts [95]. Green-synthesized ZnO NPs exhibit remarkable antimicrobial properties, high photocatalytic efficiency, and enhanced biocompatibility, making them highly valuable in biomedicine and biotechnology [96]. Plant extracts, in particular, provide an efficient and eco-friendly approach to ZnO NPs synthesis while enhancing the resulting functional properties of the NPs. For example, zinc nitrate hexahydrate has been employed as a precursor for ZnO NPs synthesis using *Vitex negundo* plant extract, demonstrating potent antibacterial activity against Gram-positive and Gram-negative bacteria [97]. Similarly, due to its rapid and cost-effective nature, there has been increasing interest in plant-based ZnO NPs synthesis. For instance, Nazir et al. successfully synthesized ZnO NPs using *Rumex dentatus* leaf extract and zinc nitrate precursors, revealing significant antibacterial activity [98]. The antimicrobial mechanisms of ZnO NPs are complex, involving several key actions: the generation of ROS, membrane disruption, and the release of Zn²⁺. These mechanisms collectively contribute to bacterial cell damage and death (Figure 1) [29,99]. Among these, the production of ROS, such as hydrogen peroxide (H₂O₂), hydroxyl radicals (•OH), and superoxide anions (O₂⁻), plays a central role. These reactive molecules induce oxidative stress within bacterial cells, damaging essential components like proteins, lipids, and nucleic acids. The resulting oxidative imbalance disrupts bacterial metabolism and compromises cellular integrity, ultimately leading to cell death [100].

Application of biosynthesized ZnO NPs against *A. baumannii*: As mentioned, *A. baumannii *poses a unique challenge due to its ability to develop MDR and form biofilms, which protect it from host immune responses and antibiotic treatments [3]. ZnO NPs have shown promise in disrupting several key virulence mechanisms in* A. baumannii*. For instance, Hou et al. fabricated ZnO NPs using *Caryophyllus aromaticus* (clove) leaf extract, which was found to stabilize the NPs with polyphenolic compounds. These ZnO NPs were highly effective against MDR* A. baumannii,* inducing bacterial cell death via DNA damage and significantly reducing biofilm production, a major contributor to bacterial resistance. This study also highlighted how ZnO NPs hindered bacterial attachment and invasion into human lung epithelial cells, showing their ability to both prevent infection and reduce biofilm viability [101]. Similarly, Droepenu et al. found that biosynthesized ZnO NPs, made from *Anacardium occidentale* (cashew) leaf extract, exhibited substantial antibacterial activity against a range of pathogenic bacteria, including *A. baumannii.* The antibacterial effect was attributed to the generation of ROS, which caused oxidative stress and disrupted bacterial cell membranes, leading to cell death [102]. Further reinforcing this, Almaary et al. demonstrated that ZnO NPs synthesized through a green method exhibited strong antibacterial activity against *A. baumannii, *with inhibition zones up to 14.96 ± 1.98 mm at a concentration of 50 µg. This antimicrobial activity was largely driven by the generation of ROS, which damaged bacterial cellular components. Moreover, synergy testing revealed that ZnO NPs enhanced the effectiveness of fosfomycin against* A. baumannii*, preventing the development of heteroresistance. This synergy suggests that bioinspired ZnO NPs could significantly improve the efficiency of existing antibiotics in treating infections, particularly in critical healthcare settings like ICUs [103]. Another study using *Azadirachta indica* (neem) extract to synthesize ZnO NPs also showed promising results against MDR *A. baumannii.* The ZnO NPs exhibited time- and concentration-dependent bactericidal activity, with over 99% bacterial cell death observed within six to eight hours at 2× minimum inhibitory concentration (MIC). The mechanism of action involved significant membrane damage, protein leakage, and bacterial cell death, primarily due to ROS generation [104]. Lastly, Yassin et al. explored ZnO NPs, synthesized from *Origanum majorana* (marjoram) leaves, which displayed impressive antibacterial effects against MDR *A. baumannii.* These ZnO NPs, with a small particle size (~12.47 nm) and negative surface charge, showed potent inhibition and demonstrated synergistic effects with colistin, with a synergism rate of 75.04%. This synergy was attributed to the disruption of bacterial membranes and oxidative stress induced by ROS generation [105].

Limitations of green synthesis: Despite its eco-friendly promise, green synthesis faces significant barriers to industrial adoption. High variability in nanoparticle quality, size, shape (spherical, hexagonal, clustered), and crystallinity, combined with low metal-ion conversion efficiencies (<50%), undermines reproducibility [106]. Feedstock consistency is further challenged by seasonal/geographic constraints and additional processing steps (drying, concentration, freezer storage), which elevate costs and energy demands [106]. A limited mechanistic understanding of the underlying biochemical pathways and the inherent variability of biological extracts further complicates process control. For ZnO NPs, sterile and precisely prepared extracts are essential to prevent contamination [107], and strict regulation of synthesis parameters (temperature, precursor concentration, time) is required to ensure batch-to-batch consistency. Moreover, surface passivation must be optimized to reduce charge recombination and enhance electron injection in solar cells. In biomedical applications, controlling nanoparticle dissolution, conducting long-term biocompatibility assessments, and performing comprehensive in vivo toxicity studies are critical to safe imaging and therapeutic use [16]. Addressing these intertwined limitations will demand interdisciplinary research and targeted innovations to unlock the full potential of green-synthesized ZnO nanomaterials.

## Conclusions

Green-synthesized ZnO NPs represent a sustainable and effective approach in combating multidrug-resistant *A. baumannii*, primarily by disrupting biofilm formation, damaging bacterial membranes, and enhancing the efficacy of conventional antibiotics. Their biogenic synthesis using plant extracts ensures environmental compatibility and reduces cytotoxic concerns often associated with chemically produced nanomaterials.

Future directions should focus on unraveling the detailed mechanisms of antimicrobial action at the molecular level, optimizing synthesis parameters for reproducibility and scale-up, and performing rigorous in vivo studies to evaluate biocompatibility and safety. Moreover, assessing the long-term stability, interaction with host cells, and resistance development potential is essential for clinical translation. Integrating ZnO NPs with existing antimicrobial regimens or their formulation into novel drug delivery platforms may further enhance therapeutic outcomes against *A. baumannii* infections.
